# Impaired colonic motility in high-glycemic diet-induced diabetic mice is associated with disrupted gut microbiota and neuromuscular function

**DOI:** 10.1530/EC-23-0078

**Published:** 2023-08-03

**Authors:** Ying Pei, Rui Wang, Wanyu Chen, Shulin Yi, Chen Huang, Shaochan Liang, Hongying Cao, Yifei Xu, Bo Tan

**Affiliations:** 1 Research Centre of Basic Integrative Medicine, School of Basic Medical Sciences, Guangzhou University of Chinese Medicine, Guangzhou, China; 2 School of Pharmaceutical Sciences, Guangzhou University of Chinese Medicine, Guangzhou, China; 3 Shenzhen Traditional Chinese Medicine Hospital, The Fourth Clinical Medical College of Guangzhou University of Chinese Medicine, Shenzhen, China

**Keywords:** diabetes, obesity, constipation, colonic motility, gut microbiota

## Abstract

**Background:**

Similar to the high-fat diet (HFD), the high-glycemic diet (HGD) contributes to the development and progression of type 2 diabetes mellitus (T2DM). However, the effect of HGD on gastrointestinal motility in T2DM and its underlying mechanisms remain unclear.

**Methods:**

Thirty C57BL/6J mice were randomly designated into the normal-feeding diet (NFD) group, HFD group, and HGD group. The plasma glucose, plasma insulin, and gastrointestinal motility were examined. Meanwhile, the tension of isolated colonic smooth muscle rings was calculated, and the gut microbiota was analyzed by 16s rDNA high-throughput sequencing.

**Result:**

After 16 weeks of HGD feeding, obesity, hyperglycemia, insulin resistance, and constipation were observed in HGD mice. Autonomic contraction frequency of the colonic neuromuscular system and electrical field stimulation-induced contractions were reduced in HGD mice. On the contrary, neuronal nitric oxide synthase activity and neuromuscular relaxation were found to be enhanced. Finally, gut microbiota analysis revealed that *Rhodospirillaceae* abundance significantly increased at the family level in HGD mice. At the genus level, the abundance of *Insolitispirillum* increased remarkably, whereas *Turicibacter* abundance decreased significantly in HGD mice.

**Conclusion:**

HGD induced constipation in obese diabetic mice, which we speculated that it may be related to neuromuscular dysmotility and intestinal microbiota dysbiosis.

## Introduction

Obesity, non-alcoholic fatty liver disease (NAFLD), and type 2 diabetes mellitus (T2DM) are thought to be caused by the Western, high-fat diet (HFD) (1). These diseases are associated with various gastrointestinal (GI) motility disorders, including gastroparesis, dyspepsia, constipation, diarrhea, and fecal incontinence (2). Previous studies found that obesity reduced colonic motility, resulting in constipation (3, 4). The intrinsic enteric nervous system (ENS) is involved in regulating colonic motility (5). HFD alters enteric neuroplasticity, neuromuscular transmission, and neuronal density (6, 7, 8). HFD reduces the density of cholinergic, nitrergic, and vasoactive intestinal peptide (VIP) neurons, which influence colonic neuromuscular function and motility via excitatory and inhibitory neurotransmitters (acetylcholine, nitric oxide, and VIP, respectively) (8, 9, 10).

For decades, research has focused on the role of the gut microbiota in ENS homeostasis and intestinal motility (13). The gut microbiota plays a pivotal role in ENS development and physiological function (14). Germ-free mice exhibited abnormalities in ENS morphology and activity (15). The interaction between gut microbiota and ENS has been demonstrated in several studies. The presence of *Lactobacillus rhamnosus* promoted cholinergic neuron development (16). *Bacteroidesthetaiotaomicron* strongly correlated with enteric neuronal density (17). Further investigations indicated that colonic dysmotility was linked to disorder in the gut microbiota (11, 12). Gut microbiota dysbiosis is considered a critical factor for the onset and complication of T2DM and obesity (18). Thus, modulation of gut microbiota homeostasis may be a potential therapeutic strategy in treating diabetic colonic motility disorders (19).

In addition to the HFD, high-glycemic diet (HGD) intake, such as rice, is strongly linked to T2DM risk in China and the Asian region (20). HGD-related foods account for nearly 30% of daily energy consumption in the Chinese population (21). In fact, an epidemiological study performed on Chinese women evidenced that high glycemic intake increased the risk of developing T2DM by 78% (22). However, the influence of HGD on diabetic GI motility remains unclear. In this study, HGD-induced obese and T2DM mice were used to investigate the impact of HGD on GI motility from the perspectives of neuromuscular function and gut microbiota.

## Materials and methods

All experimental procedures were performed according to the ethical principles and guidelines approved by the National Research Council (No. ZYD-2021-170). All mice were housed in Guangzhou University of Chinese Medicine’s Experimental Animal Center.

### Mice and nutrients

A normal fat diet (3.5 kcal/g) was purchased from Guangdong Medical Laboratory Animal Center (Foshan, China). Semi-pure HFD (60% calories as fat, 5.2 kcal/g) and customized HGD (50% calories as dextrose, 4.8 kcal/g) were purchased from MediScience Ltd (Zhuhai, Guangdong, China). The composition of normal-feeding diet (NFD), HFD, and HGD diets is shown in Table 1. Female 8 weeks old C57BL/6J mice were purchased from BesTest Bio-Tech Co., Ltd. (Zhuhai, China). Mice were housed under pathogen-free conditions in a temperature-controlled room illuminated for 12 h every day and received humane care in accordance with the study guidelines established by the Guangzhou University of Chinese Medicine Laboratory Animal Holding Care. All the experimental animals were allowed to feed and drink freely. Following acclimation for 1 week, all mice were designated into three groups, namely, the NFD group (*n* = 10), the HFD group (*n* = 10), and the HGD group (*n* = 10). After feeding with the corresponding diet for 16 weeks, mice were sacrificed by cervical dislocation after anesthesia. Colonic smooth muscle tension studies were performed according to the following protocol. The rest of the tissues were snap-frozen or fixed in formalin.

**Table 1:** The composition of NFD, HFD, and HGD diets.

NFD	HFD	HGD
Protein (≥18%)	Casein (25.84%)	Casein (20%)
Fat (≥4%)	Maltodextrin (16.15%)	Dextrose (50.5%)
Cellulose (≤5%)	Sucrose (8.89%)	Canola oil (5%)
Crude ash in feed (≤8%)	Cellulose (6.46%)	Cocoa butter (5%)
Moisture content (≤10%)	Soybean oil (3.23%)	Copha (13.1%)
Lysine (≥0.82%)	Lard (31.66%)	Cellulose (2%)
Calcium (1%-8%)	Minerals and vitamins (7.77%)	l-methionine (0.3%)
Phosphorus (0.6%–1.2%)		Calcium carbonate (1.31%)
Salt (0.3%–0.8%)		Sodium chloride (0.26%)
		Ain93 trace minerals (0.14%)
		Potassium citrate (0.25%)
		Potassium dihydrogen phosphate (0.69%)
		Potassium sulfate (0.16%)
		Choline chloride (0.25%)
		Ain93 vitamins (1%)

### Biochemical assays

Biochemical assays of plasma samples were performed according to previous research (32). Blood sample was collected from the retinal vein plexus after the mice were fasted overnight. Mice were anesthetized by isoflurane. Plasma was harvested after centrifugation (1000 ***g***, 10 min). Plasma glucose, triglyceride, and total cholesterol were determined using commercial kits from Rsbio (Shanghai, China). Plasma insulin was examined using ELISA commercial kits from IMD (Hong Kong, China). Insulin (1 U/kg) was intraperitoneally injected into mice that fasted for 6 h. Glucose kinetics of insulin tolerance test was detected at 0, 20, 60, and 120 min after injection.

### Gastrointestinal motility tests

GI motility test was performed according to previous research (33, 34). Each mouse was placed in a separate clean metabolic cage for 30 min to acclimatize. After 2 h, feces were collected, counted, and captured. For GI transit time, mice were fasted overnight and administrated oral gavage with 0.2 mL fruit-green food colorant solution (5.5% food colorant + 0.5% methylcellulose + 94% normal saline) and monitored the first green fecal pellet appearance. For colonic transit time, a 3 mm glass bead was inserted into the colon (2 cm from the anus) and monitored the bead discharge time.

### Histology

Tissues from the colon were sequentially fixed, dehydrated, paraffin-embedded, sectioned, stained with hematoxylin–eosin, and photographed and morphologically observed using an optical microscope (Olympus BX53, Olympus).

### Colonic smooth muscle tension studies (*ex vivo*)

Colonic smooth muscle tension studies were performed according to our previous research (33). Colonic smooth muscle rings were harvested and suspended in pre-cooled Kreb’s buffer (NaCl, 118 mM; KCl, 4.75 mM; MgSO_4_, 1.18 mM; NaHCO_3_, 24.8 mM; KH_2_PO_3_, 1.18 mM; CaCl_2_, 2.5 mM; and C_6_H_12_O_6_·H_2_O, 10 mM; pH, 7.4). The sample rings were equilibrated in organ baths, which were continuously perfused with Kreb’s buffer maintained at 37°C and continuously gassed with carbogen (95% O_2_ + 5% CO_2_). Each colonic ring was attached to a fixed hook and a force transducer for measuring isometric tension. The colonic rings were stretched under 0.5 g for 1 h as equilibration. Force measurements were displayed on a Powerlab strip chart recorder. After equilibration, autonomic contraction of colonic rings was recorded. Carbachol (CCh, 1 × 10^−8^–1 × 10^−5^ mol/L, Sigma) was added to the organ bath and incubated for 5 min, and each group’s contractile responses of colonic rings were recorded. After contraction induced by CCh, the colonic rings were washed with Kreb’s buffer three times and equilibrated for 1 h before the next experiment. Electrical field stimulation (EFS, 40 V, 2–32 Hz, 0.5 ms pulse duration, 10 s) stimulated the colonic rings to induce neuron-mediated relaxation and contraction with or without neuronal nitric oxide synthase (nNOS) inhibitor l-Nitro-l-Arginine Methyl Ester (l-NAME, 10 μM, Tocris Bioscience, Bristol, UK) preincubation for 5 min. The frequency–response curve was obtained at an interval of 3 min. Contractile responses of colonic rings were performed by extracellular potassium chlorides (KCl, 120 mM). Each colonic ring was weighed and recorded after blotting on filter paper at the end of the experiments. The amplitude of contraction and relaxation responses before and after stimulation were normalized by the weight and the length of tissue strip according to the formula: ((peak value − base value) (g) × length (cm) × 1.06 (mg/mm^3^) × 0.0098 (N/g))/weight (g). CCh and l-NAME were purchased from Sigma. The tension changes of colonic smooth muscle rings were displayed on the graphic recorder and data are recorded by LabChart Reader Software (LabChart Reader v8.1.21).

### Real-time polymerase chain reaction

Real-time polymerase chain reaction (RT-PCR) protocol was referred to in our previous research (35). Homogenizing tissues extracted total RNA by using EZBioscience Bio Tissue RNA purification Kit (Roseville, CA, USA) and single standard cDNA was synthesized by using a color reverse transcription kit (Roseville, CA, USA). Quantitative RT-PCR was performed with Thermo Scientific ABI-7500 RT-PCR instrument. The primers used were as follows: nNOS forward 5′-GTC AGA AGA TGT CCG CAC CAA GG-3′ and reverse 5′-TGT TCA CCT CCT CCA GCC TGT C-3′; GAPDH forward 5′-TGT GTC CGT CGT GGA TCT GA-3′ and reverse 5′-TTG CTG TTG AAG TCG CAG GAG-3′.

### Western blot

A Western blot protocol was mentioned in previous research (36). Total protein extracts were fractionated by sodium dodecyl sulfate–polyacrylamide gel electrophoresis and transferred to polyvinylidene difluoride membranes. The membranes were blocked with 5% non-fat milk in Tris-buffered saline with 0.005% Tween-20 (TBST) for 2 h at room temperature and incubated with anti-nNOS (1:10,000, BD Biosciences, San Diego, CA, USA) and anti-GAPDH (1:1000, Affinity Bioscience, Liyang, Jiangsu, China) at 4°C overnight. After washing three times with TBST, membranes were incubated with respective secondary antibodies (1:5000, Cell Signaling Technology) for 2 h at room temperature. The protein bands of primary–secondary antibody interactions were visualized with ePhoto^TM^-CL1 imaging system (Genscript Biotech, Nanjing, China). The gray value was calculated by Image J (V1.8.0.112).

### Determination of fecal microbial composition

Feces from three cages of mice (each cage containing 3–4 mice) were collected for 16s rDNA sequencing. The PCR primer was designed against the conserved region to target the variable region of the 16S rDNA gene. After 35 cycles of PCR, sequencing adapters and barcodes were added for amplification. The sequences of primers used for pre-sequencing PCR amplification were as follows: F (5'-CCT ACG GGN GGC WGC AG-3'), R (5'-GAC TAC HVG GGT ATC TAA TCC-3'). PCR amplification products were detected by 1.5% agarose gel electrophoresis. The target fragments were recovered using the AxyPrep PCR Cleanup Kit. The PCR product was further purified using the Quant-iT PicoGreen dsDNA Assay Kit. The library was quantified on the Promega QuantiFluor fluorescence quantification system. The pooled library was loaded on Illumina platform using a paired-end sequencing protocol (2 × 250 bp). Paired-end reads were assigned to samples based on their unique barcode and truncated by cutting off the barcode and primer sequence. Paired-end reads were merged using FLASH (v1.2.8) for 16S. Quality filtering on the raw reads was performed under specific filtering conditions to obtain the high-quality clean tags according to the fqtrim (v0.94). Chimeric sequences were filtered using Vsearch software (v2.3.4). After dereplication using DADA2, the feature table and feature sequence were obtained. Alpha diversity and beta diversity were calculated by QIIME2, in which the same number of sequences were extracted randomly by reducing the number of sequences to the minimum of some samples, and the relative abundance (× bacteria count/total count) is used in bacteria taxonomy. Alpha diversity and beta diversity were analyzed by QIIME2 process, and pictures were drawn by R (v3.5.2). The sequence alignments of species annotation were performed by Blast, and the alignment database was SILVA and NT-16S.

### Data analysis

All results were expressed as means ± s.e.m. Statistical analysis was performed using GraphPad Prism 8.0.1. Calculations were performed using SPSS 20.0 based on the number of individual tissue segments. Non-pairwise comparisons were performed using Student’s *t*-test. ANOVA was used in testing three or more variables for statistical significance. The correlation between the feces area and intestinal microbiota was analyzed by Pearson correlation analysis. Nonlinear and linear regression analyses were also utilized as appropriate. *P* < 0.05 was considered significant.

## Results

### Effects of HGD on obesity and T2DM development

**Figure 1:**
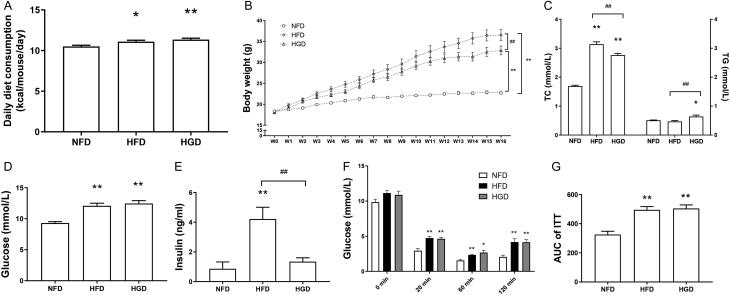
Effects of HGD on obesity and T2DM development. Daily diet consumption (A), body weight changes (B), plasma total cholesterol and triglyceride (C), fasting glucose (D), fasting insulin (E), plasma glucose kinetics of insulin tolerance test (ITT) (F), and area under curve of ITT (G) after feeding with different diets for 16 weeks. One-way ANOVA followed by Tukey's *post-hoc* tests were used for data analysis. Values are shown as mean ± s.e.m., *n*= 6–10 per group. *P < 0.05*,* **P< 0.01 vs NFD group. ^#^*P* < 0.05, *^##^P* < 0.01 vs HFD group.

Based on the higher caloric content of HGD and HFD, HGD and HFD groups ingested more calories than the NFD group (Fig. 1A, *P* < 0.05). The HGD and HFD groups exhibited greater body weight gain than the NFD group (Fig. 1B,* P* < 0.05). The HGD and HFD groups also exhibited hypercholesterolemia (Fig. 1C,* P* < 0.01). However, hypertriglyceridemia was observed only in the HGD group (Fig. 1C,* P* < 0.05). After HGD or HFD feeding, mice displayed hyperglycemia (Fig. 1D, *P* < 0.01). Interestingly, only HFD feeding induced hyperinsulinemia, and plasma insulin levels were only marginally enhanced by HGD feeding (Fig. 1E,* P* < 0.01). However, insulin sensitivity was impaired by both the HGD and the HFD (Fig. 1F and G,* P* < 0.01). Taken together, these data suggested that HGD and HFD can induce the development of obesity and T2DM.

### Effects of HGD on GI motility

**Figure 2:**
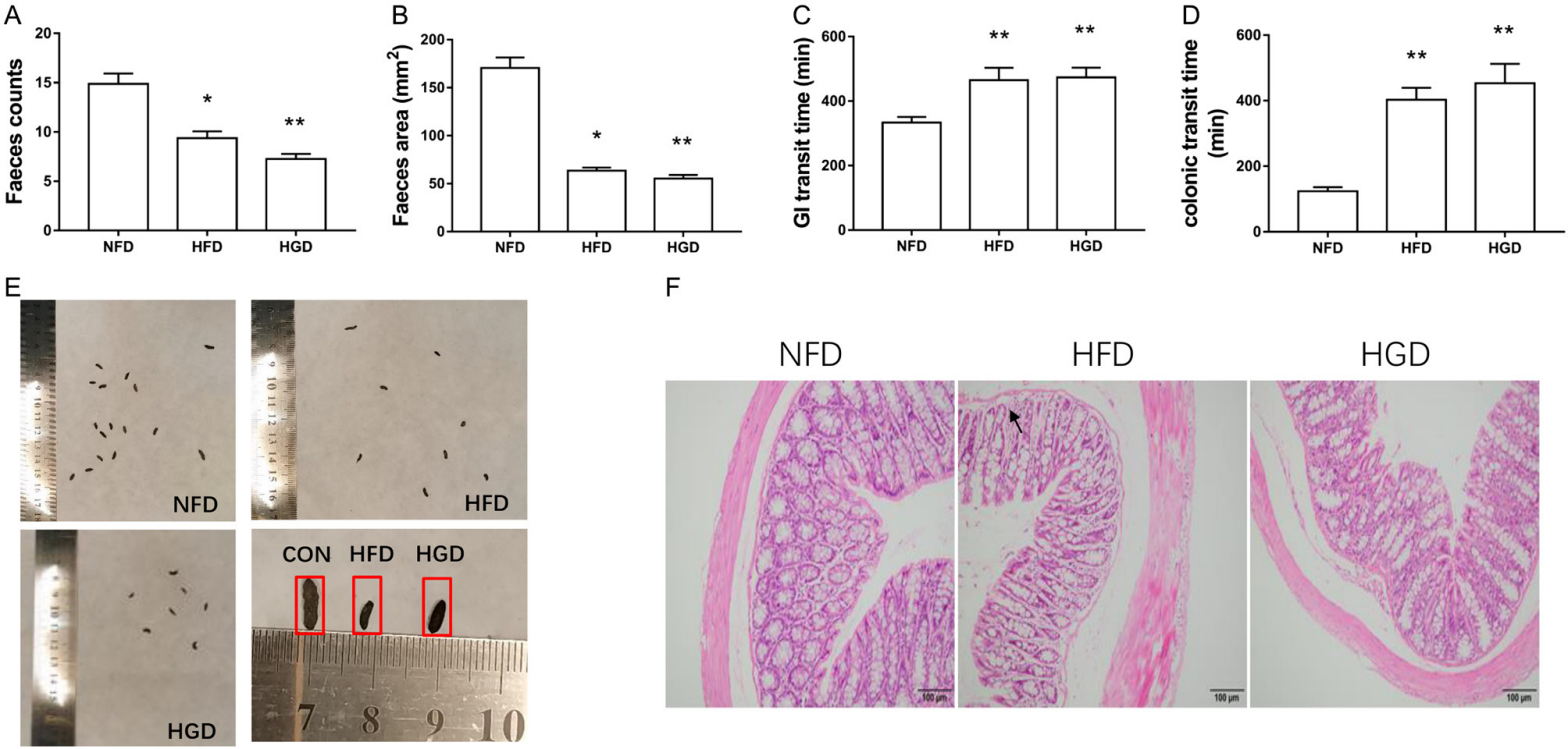
Effects of HGD on GI transit. Feces counts (A), feces area (B), GI transit time (C), colonic transit time (D), representative feces size (E), and hematoxylin and eosin staining of the colon (F) from each group after feeding with different diets for 16 weeks. One-way ANOVA followed by Tukey's *post-hoc* tests were used for data analysis. Values are shown as mean ± s.e.m., *n*= 6–8 per group. *P < 0.05*,* **P < 0.01 vs NFD group. ^#^*P* < 0.05,^##^*P* < 0.01 vs HFD group.

After T2DM development in the HGD and HFD groups, GI motility was monitored according to a previously described protocol. Fecal counts were reduced in both the HGD and HFD groups 2 h after collection (Fig. 2A,* P* < 0.05). Fecal size was also decreased in the HGD and HFD groups (Fig. 2A and E,* P* < 0.05). These data indicate that HGD and HFD can induce diabetic constipation. Subsequently, the total GI and colonic transit times were monitored. The data showed that both GI and colonic transit times increased in the HGD and HFD groups (Fig. 2C and D,* P* < 0.01). These data indicated that HGD and HFD induced constipation due to colonic motility disorders. Histomorphological staining showed no obvious lesions or necrosis in the HGD and HFD colonic tissues (Fig. 2F).

### Effects of HGD on colonic neuromuscular contraction

**Figure 3:**
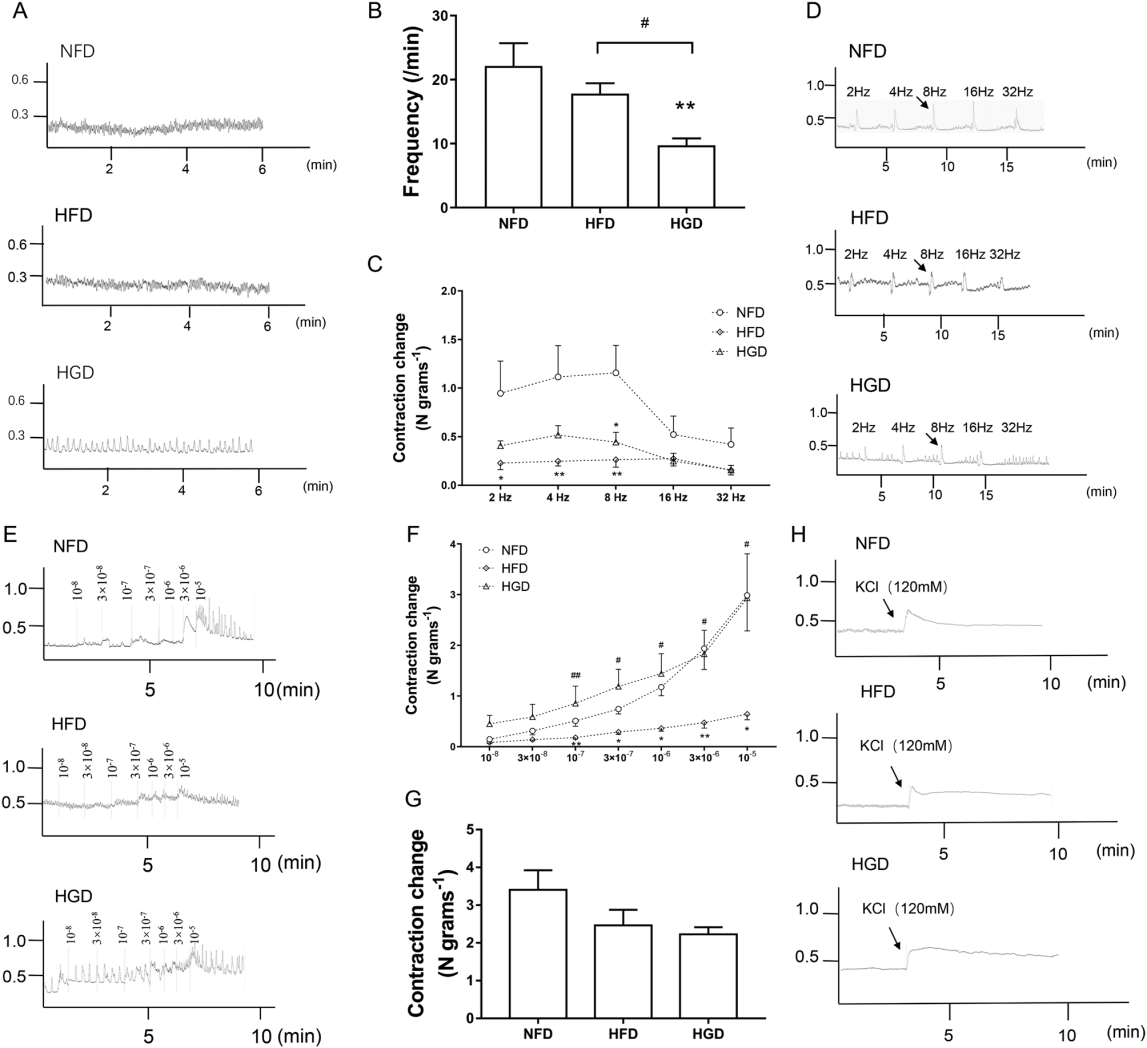
Effects of HGD on colonic neuromuscular contraction. Representative tracings showing colonic autonomic contraction (A) and summary of contraction (B). Representative tracings showing colonic contraction (D) and summary of contraction (C) induced by EFS (2–32 Hz). Representative tracings showing colonic contraction (E) and summary of contraction (F) induced by Carbachol (10^−8^–10^−5^ mol/L). Representative tracings showing colonic contraction (H) and summary of contraction (G) induced by KCl (120 mM). One-way ANOVA followed by Tukey's *post-hoc* tests or nonparametric tests followed by Kruskal–Wallis tests were used for data analysis. Values are shown as mean ± s.e.m., *n* = 6 per group. *P < 0.05*,* **P < 0.01 vs NFD group. ^#^*P* < 0.05*,* ^##^*P* < 0.01 vs HFD group.

Based on the diabetic colonic motility dysfunction found in the HGD and HFD groups, colonic neuromuscular activity was observed. After feeding with HGD, colonic autonomic contraction frequency significantly decreased (Fig. 3A and B,* P* < 0.01). In addition, EFS-induced (2–32 Hz) colonic neuromuscular contractions were decreased in the HGD and HFD groups (Fig. 3C and D,* P* < 0.05). CCh-induced (1 × 10^−8^–1 × 10^−5^ mol/L) colonic neuromuscular contractions were attenuated in the HFD group, while there was no significant change in colonic neuromuscular contraction in the HGD group compared to the NFD group (Fig. 3E and F,* P* < 0.05). In addition, colonic muscular contraction in all groups showed no obvious difference after KCl (120 mM) preincubation (Fig. 3G and H,* P* > 0.05), indicating that there was no colonic muscular structural damage in the HGD and HFD groups. Overall, these data suggested that colonic neuromuscular activity was altered by HGD and HFD. However, the effect of CCh-induced neuromuscular contractions on HGD was relatively slight.

### Effects of HGD on colonic neuromuscular relaxation

**Figure 4:**
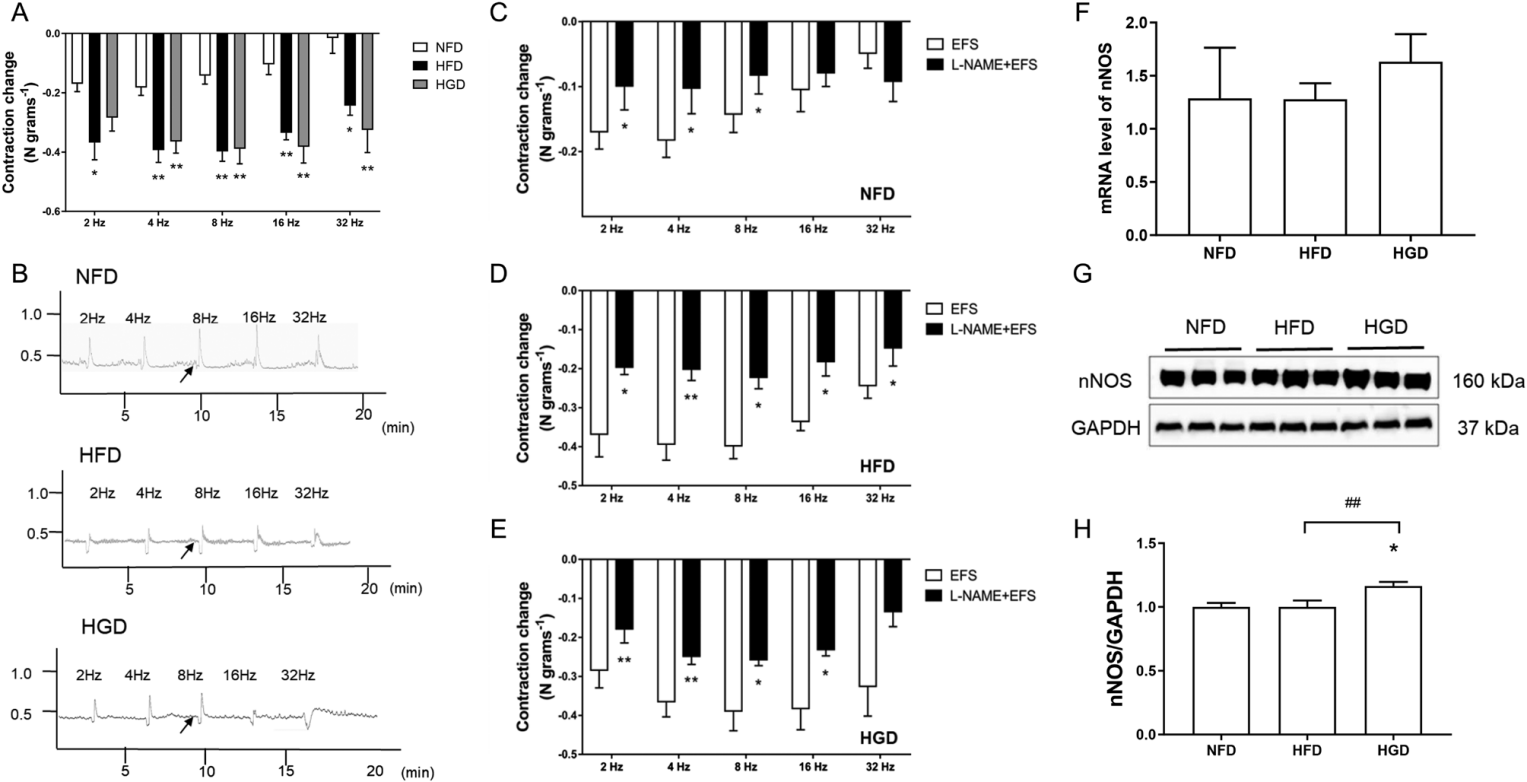
Effects of HGD on colonic neuromuscular relaxation. Representative tracings showing colonic relaxation and summary of relaxation induced by EFS (A, B). The summary of colonic relaxation changes by pretreatment with l-NAME (10 μM) and followed by EFS stimulation from NFD (C), HFD (D), and HGD (E). Colonic gene level of nNOS (F) and protein expression of nNOS (G, H) from each group. One-way ANOVA followed by Tukey's *post-hoc* tests or Independent samples *t*-tests were used for data analysis. Values are shown as mean ± s.e.m., *n* = 6 per group. *P< 0.05,**P< 0.01 vs NFD group. ^#^*P* < 0.05,^##^*P* < 0.01 vs HGD group.

Colonic neuromuscular relaxation was also measured in the HGD and HFD groups. During 4–32 Hz EFS, colonic neuromuscular relaxation distinctly increased in the HGD and HFD groups (Fig. 4A and B,* P* < 0.01). Meanwhile, EFS-stimulated colonic neuromuscular relaxation was significantly decreased after pre-incubation with l-NAME in the NFD, HFD, and HGD groups (Fig. 4C, D and E,* P* < 0.05). These data suggested that inhibitory neurotransmitters (NO) played a critical role in colonic neuromuscular relaxation in the HGD group. Next, the mRNA and protein expression levels of nNOS were measured. The mRNA levels of nNOS were similar among the three groups (Fig. 4F,* P* > 0.05). However, nNOS protein expression was significantly upregulated in the HGD group compared with the NFD and HFD groups (Fig. 4G and H,* P* < 0.05). Therefore, we postulated that enhanced colonic neuromuscular relaxation may be associated with an increased neural release of nitrergic neurotransmitters in the HGD group.

### Effects of HGD on the gut microbiota

**Figure 5:**
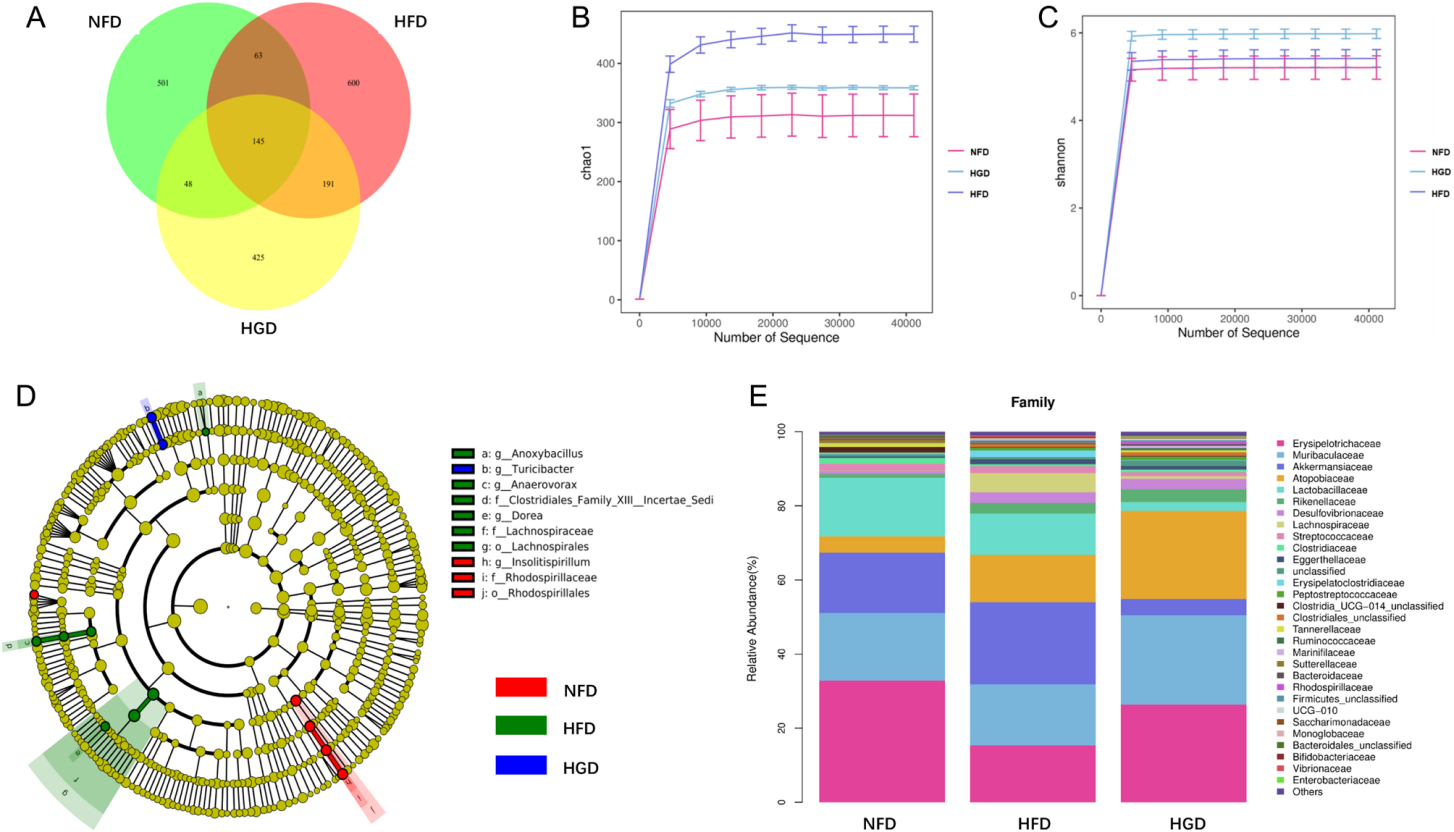

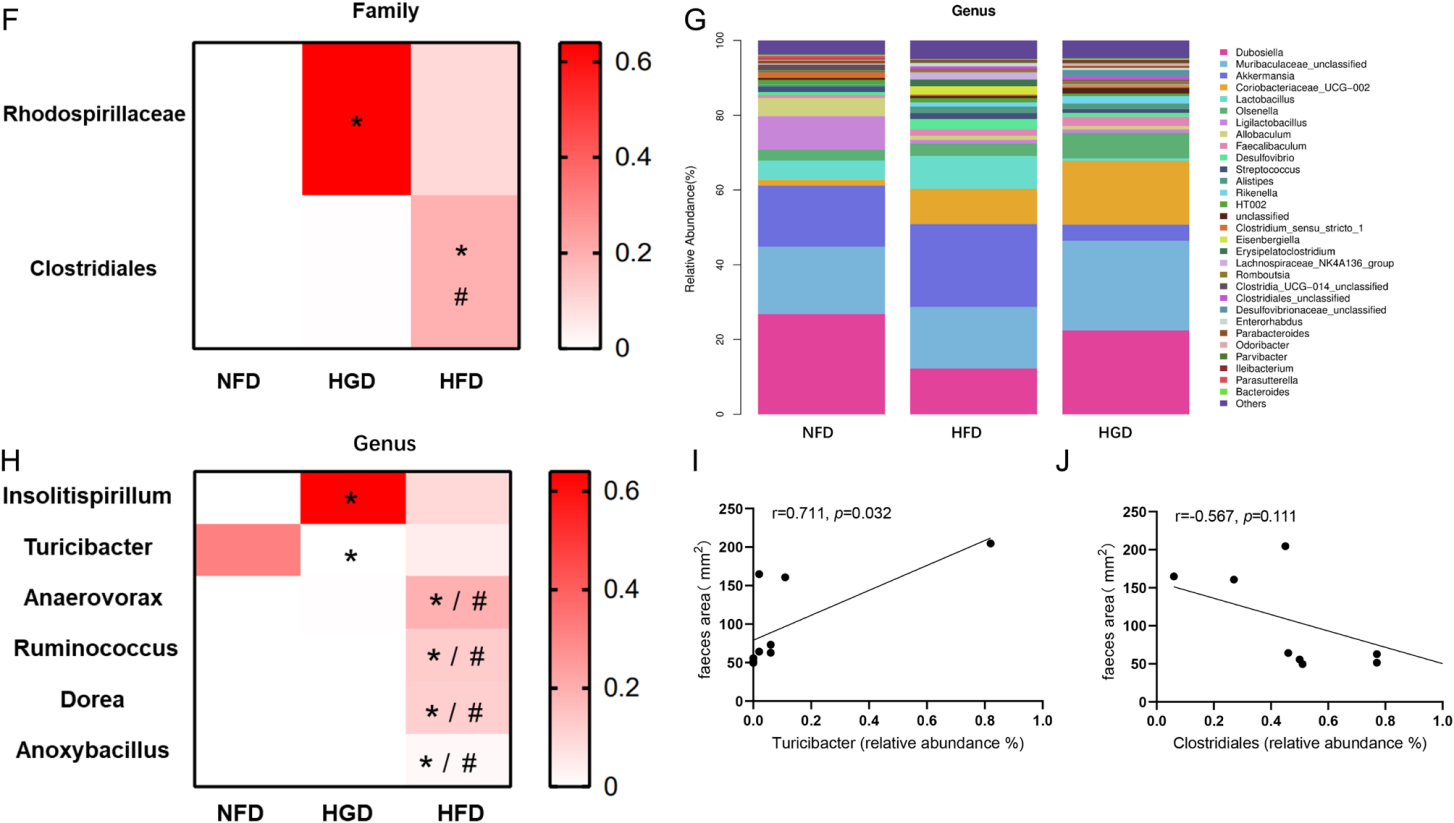
Effects of HGD on gut microbiota. Venn diagram of operational taxonomic units of each group (A). Alpha diversity analysis of gut microbiota (B, C). Cladogram of difference microbiota (D). Bacterial taxonomic profiling (E) and summary of differential bacterial flora at the family level (F). Bacterial taxonomic profiling (G) and summary of differential bacterial flora in the genus level (H). The correlational analysis between feces area and *Clostridiales* (I)*, Turicibacter* (J) at the genus level. One-way ANOVA followed by Tukey's *post-hoc* tests, Kruskal–Wallis tests, or Pearson correlation analysis was utilized for data analysis. Values are shown as mean ± s.e.m., *n* = 3 per group. *P < 0.05 vs NFD group. ^#^ *P <* 0.05 vs HGD group.

The composition of the gut microbiota was analyzed by bacterial 16S rRNA sequencing of the intestinal feces. After dereplication of unqualified sequences, clean data were obtained and analyzed. After redundancy analysis of bacterial phylotypes in each group, 145 overlapping operational taxonomic units were analyzed (Fig. 5A). The sequencing depth and diversity were covered and indicated by Chaos1 and Shannon (Fig. 5B and C). UniFrac-based principal coordinate analysis and multivariate analysis revealed that the composition of the gut microbiota in the NFD group was significantly different from that in the HGD and HFD groups (Supplementary Fig. 1, see section on supplementary materials given at the end of this article). A phyllo tree of significantly different bacterial phylotypes was determined (Fig. 5D). A detailed analysis indicated that *Clostridiales* abundance was enhanced in HFD mice at the family level, whereas *Rhodospirillaceae* abundance was increased in HGD mice at the family level (Fig. 5E and F,* P* < 0.05). At the level of genus, the *Insolitispirillum* abundance was increased in HGD mice, while the abundance of *Turicibacter* was decreased in HGD mice (Fig. 5G and H,* P* < 0.05). Additionally, the *Insolitispirillum_sp.* and *Clostridium_sp._A9* abundance was increased in HGD groups at the level of species, and the *Turicibacter_unclassified* abundance was declined in HFD groups (Supplementary Fig. 2A and B,* P* < 0.05). The correlational analysis between feces area and intestinal microbiota at the genus level showed that there was a significant positive correlation between *Turicibacter* and feces area, while *Clostridiales* and feces area had some degree of negative correlation (Fig. 5I and J). However, there was no obvious correlation between *Insolitispirillum* and feces area (Supplementary Fig. 2C). These data suggested that HGD can also alter the composition of the gut microbiota, which was possibly associated with diabetic constipation.

## Discussion

Metabolic diseases, including obesity, NAFLD, and T2DM, are accompanied by a series of GI issues, including gastroparesis, dyspepsia, constipation, diarrhea, and fecal incontinence (1, 2). HGD is one of the most critical risk factors in the development of T2DM in China and Asia (20). Since women who prefer an HGD are more susceptible to GI disorders than men (24), HGD-fed, female T2DM mice were used in this study to investigate GI motility. As a result, HGD-fed, female T2DM mice were used in this study to investigate GI motility. Hyperinsulinemia was observed in the HFD group and was not observed in the HGD group. In contrast, HGD-fed mice developed obesity, hyperglycemia, dyslipidemia, and insulin resistance. These findings suggested that HGD promotes insulin resistance and the development of T2DM. Fecal counts were reduced in both the HGD and HFD groups after 2 h. We also found that the NFD group’s feces were larger than those of the HGD and HFD groups. Thus, we concluded that HGD feeding, like HFD feeding, caused diabetic constipation. We then measured the total GI and colonic transit times to determine which segment of the GI tract was dysmotile. Surprisingly, the colonic transit time was prolonged in the HGD and HFD groups. These findings indicated that HGD, like HFD, caused diabetic colonic motility disorders (25, 26). However, there was no significant histomorphological impairment in HGD colonic tissues. We hypothesized that diabetic constipation was caused by a colonic neuromuscular disorder.

Since colonic neuromuscular motility is largely regulated by intrinsic ENS, we recorded the autonomic contraction tension of the colonic rings. Our data showed that HGD significantly reduced the colonic autonomic contraction frequency. This finding suggested that HGD feeding reduced the frequency of colonic rhythmic activity, which could be a possible mechanism underlying diabetic constipation. HFD feeding significantly reduced cholinergic neuromuscular contractions. This result has previously been demonstrated in research (26). As a result, we concluded that both the HGD and HFD altered colonic neuromuscular contraction and caused diabetic constipation, but the phenotype and mechanism of these diets differed significantly. In addition to colonic neuromuscular contraction, colonic neuromuscular relaxation also contributes to colonic motility. Focusing on the role of nitrergic neurotransmitters in colonic neuromuscular relaxation, we discovered that nitrergic neurotransmitter-mediated relaxation was important in all the three groups (27). HGD feeding increased colonic protein expression of nNOS, a potential mechanism by which HGD promotes colonic neuromuscular relaxation. Based on these findings, we hypothesized that HGD feeding reduced the frequency of colonic rhythmic activity and increases colonic neuromuscular relaxation, which may disrupt the colonic contraction-relaxation balance, resulting in diabetic constipation (23).

We also examined the potential mechanisms underlying these phenotypes based on the effects of HGD on colonic motility. According to previous studies, gut microbiota played an important role in ENS and GI motility (13, 14). Surprisingly, the most common syndrome in T2DM and metabolic diseases relates to dysbiosis of the gut microbiota (18). We performed 16S rRNA analysis of fecal samples from all three groups. The composition of the gut microbiota was altered after HFD or HGD feeding. HGD feeding increased the abundance of *Clostridium_sp._A9* at the species level. *Clostridium* growth was observed in antibiotic-induced irritable bowel syndrome mice, indicating that *Clostridium* may play a role in colonic motility (28). A previous study found that the abundance of *Clostridiales* was increased in rats with irritable bowel syndrome, indicating that the abundance of *Clostridiales* is possibly related to colonic disorder (29). In the meantime, reducing the abundance of *Clostridiales* in the gut can restore GI homeostasis and motility (29). Therefore, we hypothesized that variations in *Clostridiales* may be associated with diabetic intestinal motility disorder and constipation. In addition, *Turicibacter* abundance was notably reduced in the HGD groups at the genus level, and the correlational analysis showed that there was a significant positive correlation between Turicibacter and feces area. Previous studies have shown that *Turicibacter* was strongly associated with constipation. According to the correlation analysis, *Turicibacter* enrichment significantly improved constipation in mice and directly promoted colonic peristalsis (30). In previous research, decreasing *Turicibacter* abundance was observed in obese T2DM mice (31). These above findings suggested that HGD altered colonic activity, which may be related to intestinal microbiota dysbiosis, especially the increased *Clostridiales* abundance and decreased *Turicibacter* abundance.

In conclusion, HGD induced constipation in obese T2DM mice, but the underlying mechanisms were not entirely consistent with HFD-induced diabetic constipation. HGD-induced diabetic constipation may be mainly related to colonic neuromuscular motility disorders and intestinal microbiota dysbiosis.

## Supplementary Materials

Supplementary Fig 1. UniFrac-based PCoA (A) and Multivariate analysis (B) of fecal samples from NFD, HGD and HFD, n=3 per group.

Supplementary Fig 2. Bacterial taxonomic profiling (A) and summary of differential bacterial flora in the species level (B). The correlational analysis between feaces area and Insolitispirillum (C) at the genus level. Values are shown as mean ± SEM, n=3 per group. * p <0.05, versus NFD group. #p<0.05 versus HGD group.

## Declaration of interest

The authors have no conflict of interest to declare.

## Funding

This work was funded by the National Natural Science Foundation of Chinahttp://dx.doi.org/10.13039/501100001809 (grant number 81973586); Key Project of Department of Education of Guangdong Provincehttp://dx.doi.org/10.13039/501100010226 (grant number 2022ZDZX2019); ‘Double First-class’ and High-level University Discipline collaborative innovation team project of Guangzhou Universityhttp://dx.doi.org/10.13039/501100014881 of Chinese Medicine (grant number 2021xk37). National Science Foundationhttp://dx.doi.org/10.13039/100000001 of China (grant number 82204734); Project of Administration of Traditional Chinese Medicine of Guangdong Province of China (grant number 20213015); Shenzhen Science and Technology Plan Project (grant number JCYJ20180302173834208).

## Data availability statement

The data that support the findings of this study are available on request from the corresponding author.

## Author contribution statement

Y Pei, Y Xu, and B Tan designed the research protocol. Y Pei, W Chen, C Huang, S Yi, and S Liang implemented the research protocol. Y Pei, R Wang, and H Cao analyzed data. R Wang and W Chen wrote the manuscript. Y Pei, R Wang, Y Xu, H Cao, and B Tan revised the manuscript. All authors read and approved the final study.
