# Supplementary figures and images for: Impaired colonic motility in high-glycemic diet-induced diabetic mice is associated with disrupted gut microbiota and neuromuscular function
Source: Endocr Connect. 2023 Aug 3;12(9):e230078. doi: 10.1530/EC-23-0078 (PMC10448599; doi:10.1530/EC-23-0078)

A

PCoA plot

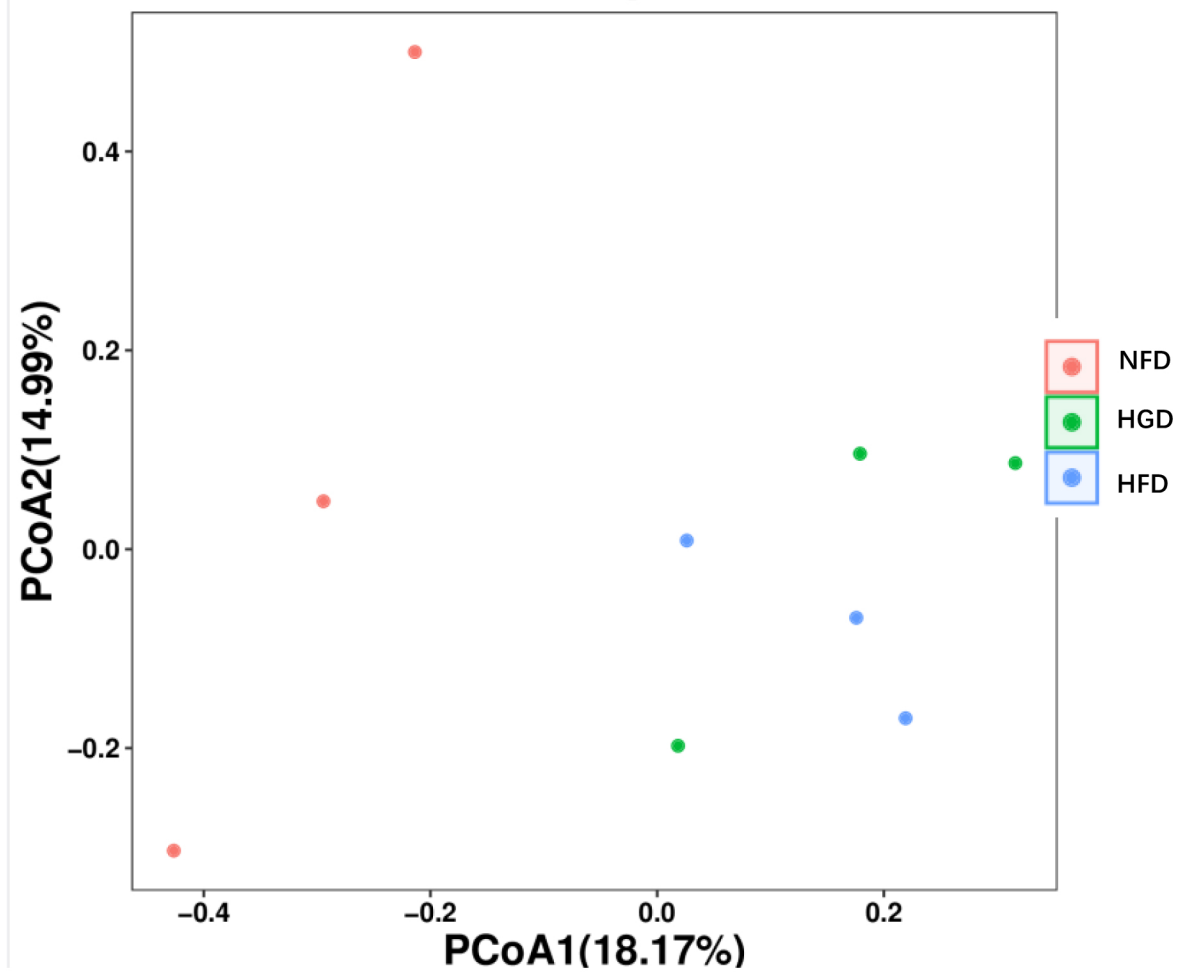

B

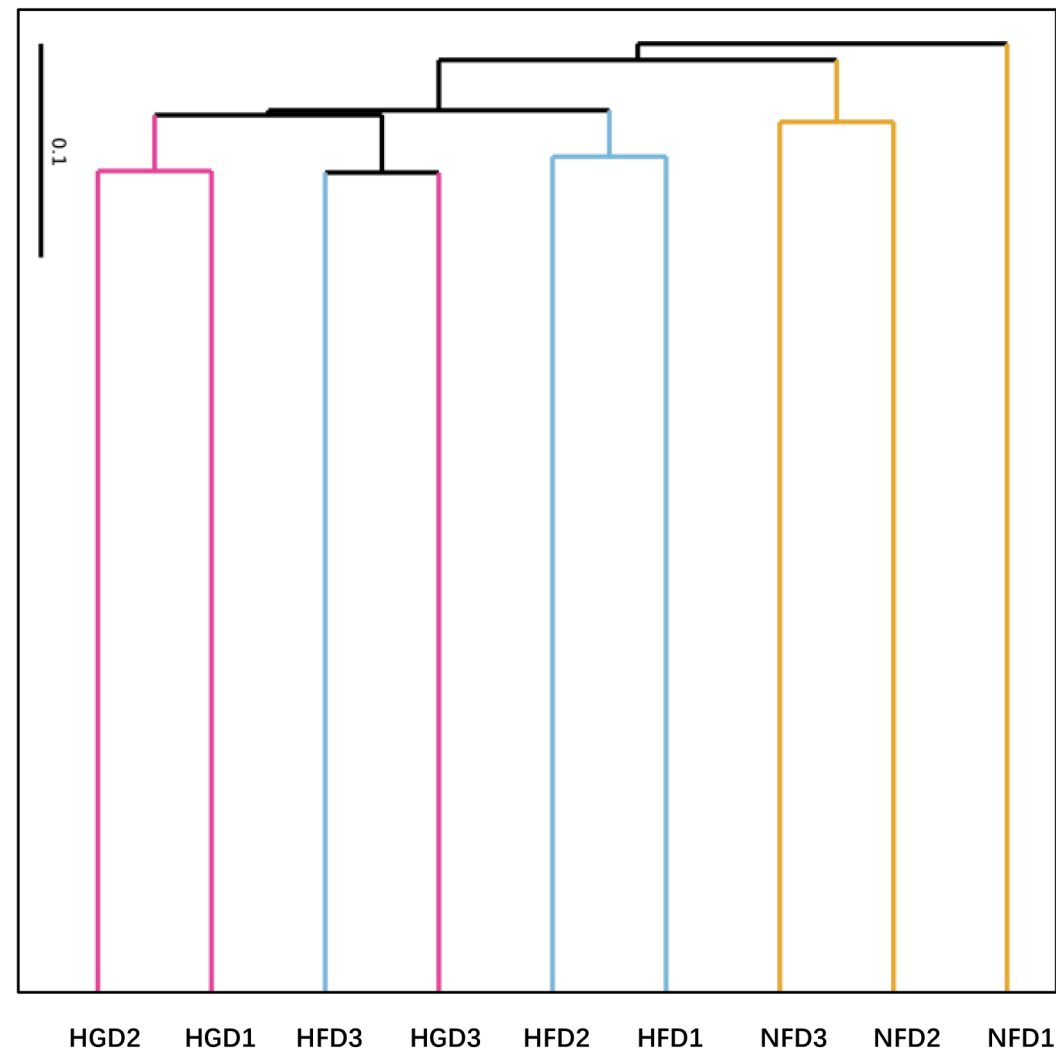

Supplement: Supplementary Fig 1. UniFrac-based PCoA (A) and Multivariate analysis (B) of fecal samples from NFD, HGD and HFD, n=3 per group. [file supplementary_figure_1.pdf]

A

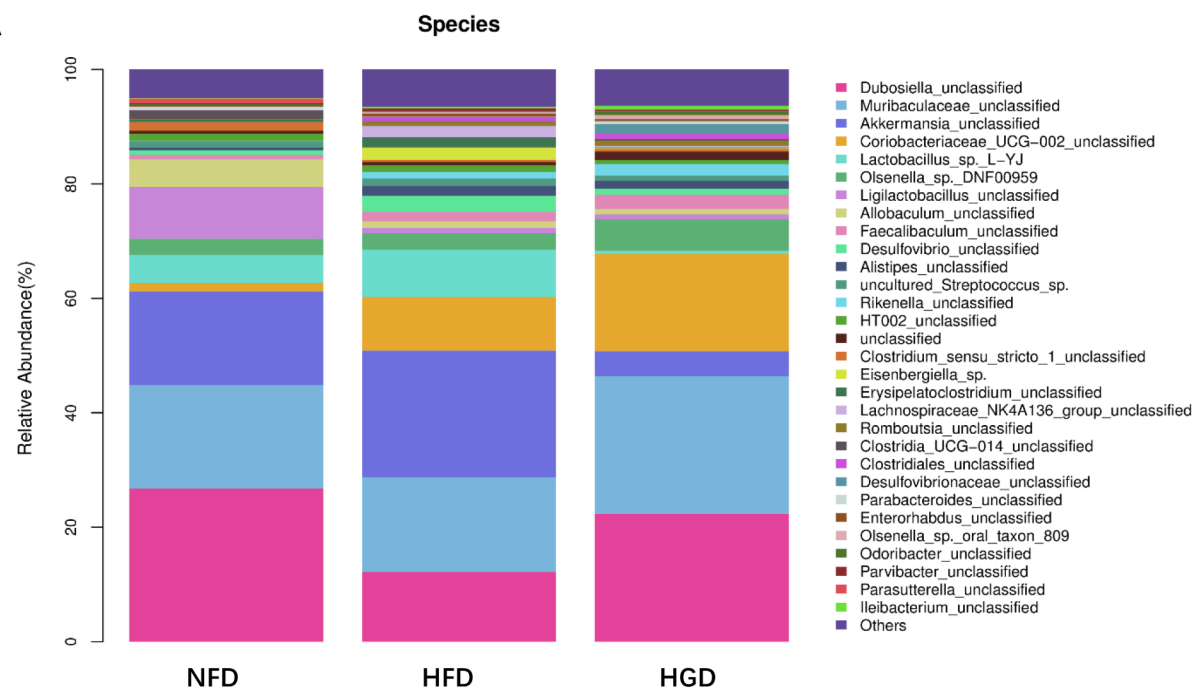

B

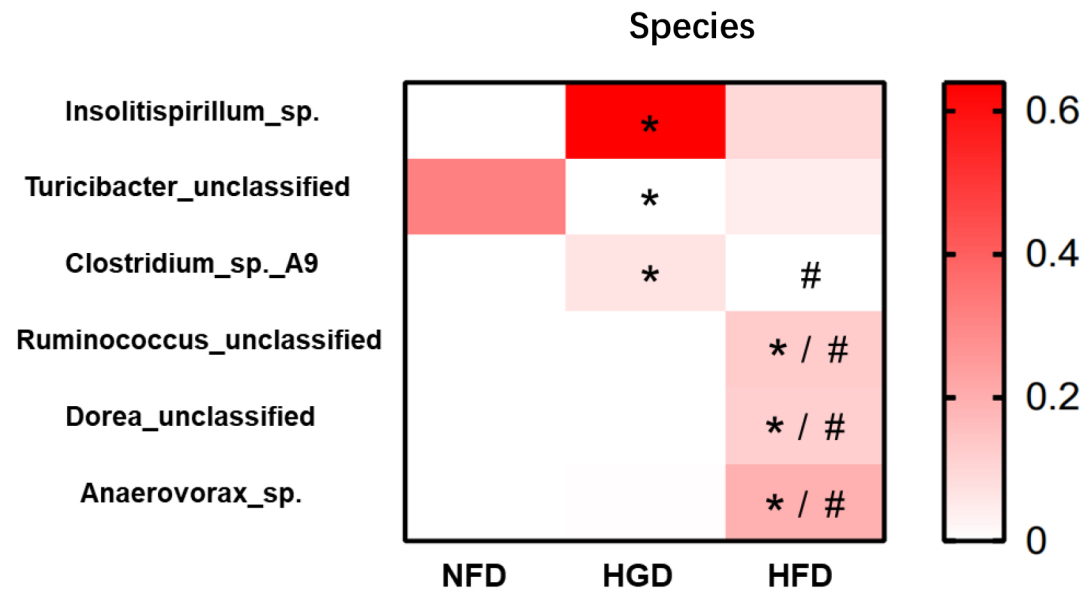

C

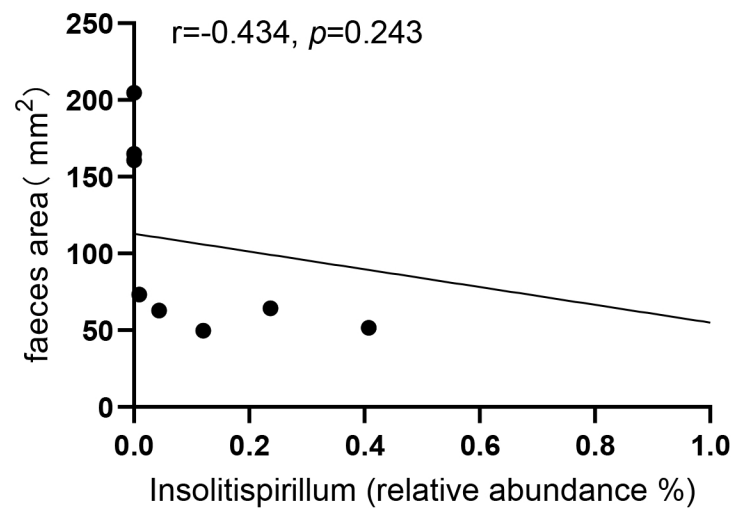

Supplement: Supplementary Fig 2. Bacterial taxonomic profiling (A) and summary of differential bacterial flora in the species level (B). The correlational analysis between feaces area and Insolitispirillum (C) at the genus level. Values are shown as mean ± SEM, n=3 per group. * p <0.05, versus NFD group. #p<0.0 [file supplementary_figure_2.pdf]
